# Harnessing phytomicrobiome signaling for rhizosphere microbiome engineering

**DOI:** 10.3389/fpls.2015.00507

**Published:** 2015-07-14

**Authors:** Liliana Quiza, Marc St-Arnaud, Etienne Yergeau

**Affiliations:** ^1^Energy, Mining and Environment, National Research Council Canada, MontréalQC, Canada; ^2^Institut de Recherche en Biologie Végétale – Jardin Botanique de Montréal and Université de Montréal, MontréalQC, Canada

**Keywords:** rhizosphere, signaling, beneficial microorganisms, agriculture, plant–microbe interactions

## Abstract

The goal of microbiome engineering is to manipulate the microbiome toward a certain type of community that will optimize plant functions of interest. For instance, in crop production the goal is to reduce disease susceptibility, increase nutrient availability increase abiotic stress tolerance and increase crop yields. Various approaches can be devised to engineer the plant–microbiome, but one particularly promising approach is to take advantage of naturally evolved plant–microbiome communication channels. This is, however, very challenging as the understanding of the plant–microbiome communication is still mostly rudimentary and plant–microbiome interactions varies between crops species (and even cultivars), between individual members of the microbiome and with environmental conditions. In each individual case, many aspects of the plant–microorganisms relationship should be thoroughly scrutinized. In this article we summarize some of the existing plant–microbiome engineering studies and point out potential avenues for further research.

## Introduction

Virtually every plant part is colonized by microorganisms, including bacteria, archaea, fungi, collectively designated as the plant–microbiome or phytomicrobiome. Depending on the plant part it colonizes, the phytomicrobiome is often referred to as endophytic (inside plant parts), epiphytic (on aboveground plant parts), or rhizospheric (in the soil closely associated to the roots) (Kowalchuk et al., 2010; Lakshmanan et al., 2014). Microorganisms are a key component of the plant, often inextricable from their host and the plant–microbiome is thought to function as a metaorganism or holobiont (Bosch and McFall-Ngai, 2011; Vandenkoornhuyse et al., 2015). The biomass and composition of the microbiome strongly affects the interactions between plants and their environments (Ryan et al., 2009). The rhizosphere harbors a largely increased bacterial abundance and activity, not only as compared to other plant compartments (Smalla et al., 2001; Kowalchuk et al., 2002), but also when compared to bulk soil. However, bacterial diversity in the rhizosphere is generally lower than in the bulk soil (Marilley and Aragno, 1999) and microbial community composition is very different (Smalla et al., 2001; Kowalchuk et al., 2002; Griffiths et al., 2006; Kielak et al., 2008; Bulgarelli et al., 2012; Peiffer et al., 2013), suggesting a strongly selective environment. As the microbial density, diversity, and activity in the endosphere (the microbial habitat inside both above- and belowground plant organs) and phyllosphere (the aboveground plant surfaces) are generally lower than in the rhizosphere, the focus of this contribution will be mainly on the rhizosphere.

**FIGURE 1:**
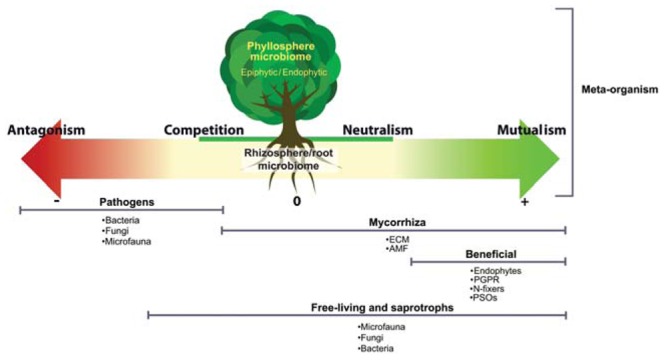
**The different interactions taking place within the plant–microbiome meta-organism.** A vast spectrum of microorganisms are involved in these interactions: ectomycorrhiza (ECM), arbuscular mycorrhizal fungi (AMF), plant growth promoting rhizobacteria (PGPR), phosphate-solubilizing organisms (PSOs), endophytes, epiphytes, and microfaunal organisms.

There is ample evidence that shows that the plant–microbe relationship is critical to health, productivity and the overall condition of the plant (Baudoin et al., 2003; Chaparro et al., 2012, 2014; Marasco et al., 2012; Adesemoye and Egamberdieva, 2013; Ziegler et al., 2013; Glick, 2014). There are different kinds of interactions between plants and microbes, spanning the whole spectrum from beneficial to pathogenic, and the outcome of the interaction between a plant and a microbe can vary among this spectrum depending on plant species, nutrient conditions, etc. (**Figure 1**). The goal of plant–microbiome engineering is to push this interaction toward enhanced beneficial outcomes for the plant. Many microbially mediated functions are important to enhance beneficial outcome, including nutrient cycling, mineralization of soil organic matter, induction of disease resistance and response to abiotic stresses such as drought and salinity (Marasco et al., 2012; Zolla et al., 2013). The plant–microbiome interactions are complex and often depend on plant species/cultivar, soil type and environmental conditions such as biotic/abiotic stress, climatic conditions, and anthropogenic effects. Different soils as well as different environmental stresses (e.g., nutrient deficiencies, metal toxicity, pathogen attack, etc.) have been shown to trigger plant-species-dependent physiological responses and consequently different exudation patterns (Bais et al., 2006; Oburger et al., 2013). Microbes in the rhizosphere can also influence the plant exudation, as for example, when antimicrobial-resistant *Pseudomonas* block the production of plant antimicrobial compounds (Hartmann et al., 2009). One interesting avenue for rhizosphere microbiome engineering is to harness these variations in exudation patterns to enhance the beneficial rhizosphere microbiome.

## Signaling in the Rhizosphere

A variety of direct and indirect interactions take place in the rhizosphere such as plant–plant, microbe–microbe, and plant–microbe, as well as the interaction with the other eukaryotic micro-, meso-, and macro-soil inhabitants (Tarkka et al., 2008; De-la-Peña et al., 2012). In view of the complexity of these interactions, knowledge of the chemical communication between all members is essential to unravel how microbial populations coordinate their behavior and interact with the plant roots. Numerous literature reviews have addressed the many different molecules and mechanisms that coordinates the establishment of specific symbiotic interactions in the rhizosphere with the potential to enhance plant growth and productivity (Mabood et al., 2008; Pieterse et al., 2009; Berendsen et al., 2012; Miller and Oldroyd, 2012; Sugiyama and Yazaki, 2012; Bakker et al., 2013; Morel and Castro-Sowinski, 2013; Oldroyd, 2013). However, the understanding of the interactions between the plants and the microbiome as a whole is still rudimentary as the diversity of organisms, molecules, and mechanisms of interaction involved is staggering. Nevertheless, the signaling compounds that make part of this complex rhizosphere interaction have the potential to improve plant functions of interest if understood and harnessed.

Plants have been found to release 5–20% of net photosynthetically fixed C into the rhizosphere (Marschner, 1995). These rhizodeposits include inorganic (CO_2_ from cell respiration and H+ eﬄux) and a variety of complex organic compounds like sloughed-off cells and tissue, intact root border cells, mucilage (polysaccharides) and proteins, all of them classified as high molecular weight compounds. Also, part of the rhizodeposits are the insoluble and soluble low molecular weight (LMW) organic compounds, collectively known as root exudates, which are actively or passively released by growing roots. Root exudates can be classified in different classes such as sugars, amino acids, and amides, organic acids, as well as aromatic and phenolic acids (Bais et al., 2006, 2008; De-la-Peña et al., 2012; Oburger et al., 2013; Zhang et al., 2015). This complex cocktail of root-secreted molecules mediate the interactions occurring in the rhizosphere (Bakker et al., 2012; Berendsen et al., 2012; Lakshmanan et al., 2014; Qiu et al., 2014) and acts as chemical attractants and repellants to shape the root microbiome (Walker et al., 2003; Berendsen et al., 2012; Ellouze et al., 2012). From the plant point of view, the goal of shaping the rhizosphere microbiome is to attract preferred partners like plant growth promoting microorganisms through the exudation of specific carbon compounds that can be used as feed and to deter pathogens or unwanted competitors for nutrients through the exudation of antimicrobial compounds such as volatiles or proteins (Bais et al., 2006; Lioussanne et al., 2008; Badri et al., 2009; Hartmann et al., 2009; De-la-Peña et al., 2012; Dangl et al., 2013). Plant exudates are also involved in coping with herbivores, encouraging beneficial symbioses, changing the chemical and physical properties of the soil, and inhibiting the growth of competing plant species (Ping and Boland, 2004; Badri et al., 2009; Morel and Castro-Sowinski, 2013).

The quality and amount of root exudates are highly dynamic in time and space and they depend on the plant species/cultivars, the physiological stage of the plant (Chaparro et al., 2013, 2014), presence or absence of neighbors, plant nutritional status, mechanical impedance (Bengough and Mullins, 1990), sorption characteristics of the soil, and the microbial activity in the rhizosphere (De-la-Peña et al., 2012; Oburger et al., 2013). Plant productivity, nutrient allocation, and tissue chemistry can also vary significantly depending on the identity of neighboring individuals, suggesting that the effects of a given plant host on the soil microbiome may be substantially mediated by the community context of that host (Bakker et al., 2012, 2013). Although very complex and still not well understood, exudation has therefore the potential to highly influence plant performance, health, and competiveness. Some studies have started analyzing the composition of plant root exudates (Phillips et al., 2008; Oburger et al., 2013; Ziegler et al., 2013), but the diversity of the compounds involved and the complexity of the soil matrix makes comprehensive analysis difficult to perform.

Many microorganisms also secrete signaling compounds in the rhizosphere. According to their functions and characteristics, these compounds have been categorized into: phytohormones, extracellular enzymes, organic acids, surface factors [compounds recognized by the host plant that activate an immune response via high-affinity cell surface pattern-recognition receptors (PRR), e.g., flagellins and lipopolysaccharides in *Pseudomonas* (Ping and Boland, 2004; Dangl et al., 2013)], antibiotics and volatile signals. Plant-associated bacteria produce and utilize diffusible quorum sensing (QS) molecules (e.g., *N*-acyl-homoserine lactones, AHLs) to signal to each other and to regulate their gene expression (Berendsen et al., 2012). Bacterially produced AHLs have been shown to affect root development of *Arabidopsis* (Ortiz-Castro et al., 2008) and have been suggested to elicit a phenomenon known as induced systemic resistance (ISR) which allows the plants to endure pathogen attacks that could be lethal without the presence of these bacterial factors. The effect of this mechanism is systemic, e.g., root inoculation with many different plant growth promoting rhizobacteria (PGPR) such as *Pseudomonas, Burkholderia*, and *Bacillus* sp. results in the entire plant being non-susceptible to pathogens (Schuhegger et al., 2006; Choudhary et al., 2007; Tarkka et al., 2008), further highlighting the importance of AHLs in cross-kingdom signaling in the rhizosphere. Plant can also exploit this microbial communication system to manipulate gene expression in their associated microbial communities. For instance, some plant-associated bacteria have LuxR-like proteins that are stimulated by plant signals (Soto et al., 2006; Ferluga and Venturi, 2009). Certain bacteria have the capacity to quench signals by degrading various plant- and microbial-produced compounds in the rhizosphere such as quorum sensing signals (Tarkka et al., 2008) and other compounds, like ethylene, that might have negatively affected plants (Bais et al., 2006).

Many of the plant response implicate the intervention of the plant immune (system systemic acquired resistance or SAR) consisting of two interconnected levels of receptors, one outside and one inside the plant cell, that govern recognition of microbes and response to infections. The first level of the plant immune system is governed by extracellular surface PRRs that are activated by recognition of evolutionarily conserved pathogen or microbial-associated molecular patterns (PAMPs or MAMPs). Activation of PRRs leads to intracellular signaling, transcriptional reprogramming, and biosynthesis of a complex output response. This response limits microbial colonization (Dangl et al., 2013) and shapes the soil microbial community in the rhizosphere by selective feeding of beneficial microorganisms and by excreting substances with antimicrobial potential such as root volatiles or root proteins which acts as the primer barriers of plant defense (Bais et al., 2006; Badri et al., 2009; Hartmann et al., 2009; De-la-Peña et al., 2012; Dangl et al., 2013).

## How Can we Engineer the Rhizosphere Microbiome?

The efforts to elucidate rhizosphere interactions have often been directed to the potential of single plant root exudates to affect single bacteria or fungi. The clear limitation of this type of approach is the removal of the organism from any context that would give relevance to interspecies interactions. The high diversity of the root- and microbe-secreted molecules involved in rhizosphere interactions suggests that studying the direct influence of a single compound on the microbiome might be impossible or not realistic in nature (Ziegler et al., 2013). Rhizosphere engineering in the environment is still a major challenge even though some studies showed some promising results (**Tables 1–3**). A vast diversity of approaches based on inter-kingdom communication has been utilized in laboratory, greenhouse, and field experiments in order to favor beneficial services to the plants while minimizing inputs requirements. Three potential routes are suggested below in **Table 1**, the microbiome approach, **Table 2**, the plant approach, and **Table 3**, the meta-organism approach. In these tables, we review more generally rhizosphere engineering efforts, but below we will more specifically focus on rhizosphere microbiome engineering studies that took advantage of signalisation channels.

**Table 1:** Selected microbiome-based methods used to engineer the rhizosphere microbiome.

Method	Mechanisms/examples	Advantages	Disadvantages	Reference
Application of microbial inoculants (biofertilizers).	Plant growth promoting rhizobacteria (PGPR), Nitrogen fixing Rhizobia Arbuscular Mycorrhizal Fungi (AMF), Ectomycorrhiza (ECM) Endophytes	Enhance plant disease control and plant performance. Phytohormone production. Increase plant immunity inducing defense mechanisms system systemic acquired resistance – induced systemic resistance (SAR – ISR) in the plant. Improve soil fertility by granting access to nutrients. Promote nodulation and nitrogen fixation. Fill empty niche spaces increasing community evenness. Induction of suppressive soils.	Establishment of very high population densities immediately after inoculation, but densities decline over time and distance from the inoculum source. Potential risks associated with the release into the environment. Unknown effect over native microbial communities.	Bünemann et al. (2006), Mabood et al. (2008), Ryan et al. (2009), Taghavi et al. (2009), Friesen et al. (2011), Bakker et al. (2012), Chaparro et al. (2012), Morel and Castro-Sowinski (2013)
	Recombinant strains.	Transfer of specific genes by horizontal gene transfer (HGT) inducing the expression of beneficial functions. Adaptation and competence development (resistance, resilience, stability).	Loss of the gene of interest within the time. Potential risks associated with the release into the environment (recombinant strains). Unpredictable or undesired results related to the HGT.	Lynch et al. (2004), Mercier et al. (2006), Ryan et al. (2009)
Disruption of microbial communities to facilitate introduction of beneficial microorganisms	Imposition of mechanical or chemical disturbances: tillage, fungicides, antibiotics, etc.	Easier to establish exogenous communities.	Induce soil vulnerability.	O’Connell et al. (1996), Brussaard et al. (2007), Bakker et al. (2012)

**Table 2:** Selected plant based methods used to engineer the rhizosphere microbiome.

Method	Mechanisms/examples	Advantages	Disadvantages	Reference
Plant breeding and cultivar selection.	Enhancing exudates production of stimulatory or inhibitory factors.	Influence microbial populations by inhibiting or enhancing the growth of selected microbial members of the rhizosphere community. It does not require change in infrastructure or management in the field.	Need for deeper knowledge on the impact of diversity, quantity, and consistency of exudation shaping the microbiome. There is no control over the variability across environments, soil types, and microbial communities. There is no breeding program that evaluates plant lines for interactions with the soil microbiome.	Lemanceau et al. (1995), Hartmann et al. (2009), Ryan et al. (2009), An et al. (2011), Bakker et al. (2012)
	Alteration of plant resistance to disease and environmental factors.	Improved ability to resist to adverse environmental conditions (climatic, edaphic, and biological).	May produce unexpected or undesirable outcomes.	O’Connell et al. (1996), Lynch et al. (2004)
	Selection of mutants with enhanced capacity to form mutual symbiosis	Improved access to nutrient	Could be deleterious under high nutrient conditions Higher percentage of carbon allocated to symbionts	Solaiman et al. (2000)
Genetic modification: change in the amount and/or quality of the organic exudates, signal molecules, and residues entering the soil.	Engineering plants to produce exudates to favor specific diversity or beneficial services.	Plant induction of microbiome beneficial functional traits such as nodulation, siderophore, anti-microbial, anti-fungal, or biological control compounds. Improve resistance to adverse environments. Use in bioremediation of toxic compounds.	Inter-species plant-microbe gene transfers. When a desired trait has been engineered successfully into a plant, the compounds might be rapidly degraded, inactivated in the soil, or the rate of exudation might be too small to influence the rhizosphere as predicted.	Truchet et al. (1991), Downie (1994), O’Connell et al. (1996), Zupan et al. (2000), Brussaard et al. (2007), Broeckling et al. (2008), Bakker et al. (2012), Sharma et al. (2013)
	Engineering plants to produce exudates to modify soil properties (acidic pH, anion eﬄux from roots).	Improve plant growth at low pH, salinity resistance, and water deficit. Enhance plant Al^3+^ resistance. Improve ability to acquire insoluble P. Larger roots, longer root hairs, and greater shoot biomass.	Enzyme activities do not necessarily lead to anion accumulation and enhanced eﬄux, and suggest that metabolic or environmental factors can influence the effectiveness of this approach. The gene TaALMT1 (malate release in the rhizosphere) needs to be activated by Al^3+^.	Koyama et al. (1999), Koyama et al. (2000), Tesfaye et al. (2001), Anoop et al. (2003), Li et al. (2005), Brussaard et al. (2007), Delhaize et al. (2007), Gévaudant et al. (2007), Yang et al. (2007), Ryan et al. (2009)
	Generation of transgenic plants producing quorum sensing signal molecules *N*-acyl-homoserine lactone (AHL).	May lead to an increase in plant disease resistance by blocking communication among members of the plant-associated bacterial community.	Blocking communication among members of the beneficial plant associated bacterial community.	Teplitski et al. (2000), Savka et al. (2002), Bakker et al. (2012)
	Engineering plants to produce an enzyme responsible for degradation of the quorum sensing signal (lactonases, acylases).	Prevention of bacterial infection.	Rhizosphere populations would be able to capture and stably integrate transgenic plant DNA, in particular antibiotic resistance genes used in the selection of successful transgenic plants.	Dong et al. (2001), Braeken et al. (2008), Zhang et al. (2015)

**Table 3:** Selected meta-organism-based methods and other complementary methods used to engineer the rhizosphere microbiome.

Method	Mechanisms/examples	Advantages	Disadvantages	Reference
**Meta-organism-based**				
Selecting and managing complementary plants and microbiomes	Crop Rotation	Induction of suppressive soils by managing soil diversity. Higher level of nutrients cycling and increase of organic carbon. Improvement of physico-chemical soil characteristics.	Mechanisms are not fully understood	Mazzola (2002, 2007), Ryan et al. (2009)
Engineering plants to produce one or more compounds and engineering the inoculated bacteria to degrade these compounds.	Opine producing plants co-inoculated with opine utilizing bacteria	Establishing a direct trophic link between the two partners of the interaction.		Savka and Farrand (1997), Dessaux et al. (1998), Savka et al. (2002)
**Other methods**				
Agricultural Inputs	Mineral fertilizers: urea, ammonium nitrate, sulfates, and phosphates.	Indirectly enhance soil biological activity via increases in system productivity, crop residue return, and soil organic matter.	N fertilization generates soil acidification and P fertilization affect root colonization of AMF.	Savka et al. (2002), Bünemann et al. (2006), Mazzola (2007)
	Organic fertilizers: animal manures, composts, and biosolids.	Increase in soil organic matter increase soil biological activity (organic fertilizers).	Biosolids: possible presence of toxic substances for the soil microflora. Inability to predictably reproduce compost composition.	

### The Microbiome Route

**FIGURE 2:**
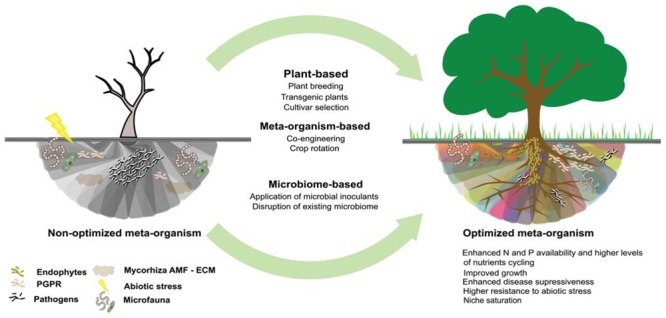
**Different approaches to rhizosphere microbiome engineering used to bring the microbiome from a low diversity and vulnerable state, with limited functions and productivity to a diverse and resilient state with high functional redundancy and consistent functioning across variable environments and increased resistance to pathogen invasion**.

Many of the bacteria in the rhizosphere are currently unable to grow in the laboratory and culture-based methods are often inadequate for qualitative analysis of the rhizosphere microbiome. As a consequence, culture-independent approaches such as metagenomics, metatranscriptomics, metaproteomics, and metabolomics have been the approaches of choice when investigating the rhizosphere microbiome (De-la-Peña et al., 2012; Bell et al., 2014a,b; Yergeau et al., 2014; Zhang et al., 2015). However, many rhizosphere microbiome engineering approaches require having microbial isolates at hand, and further efforts to increase the cultivability of rhizosphere microorganisms will be needed. Even though cultured microorganisms show certain functional capacity of their own, it is not clear yet how they behave once they are introduced in a new environmental niche as in some cases they have been shown to be out-competed by the indigenous microbial population (Ryan et al., 2009). The persistence and functionality of these isolates after inoculation need to be further assessed in order to ascertain positive impacts when used as a strategy to manipulate the rhizosphere microbiome (Stefani et al., 2015). Colonization and dominance of specific microbial species in the rhizosphere is critical for both pathogenic and beneficial soil microbes and will have an impact on disease incidence. Although a general increase in the abundance of microbes is always noted in the rhizosphere as compared to bulk soil, the community structure and functional consequences associated to this increase are poorly understood (Bais et al., 2006; Bakker et al., 2012). An increase in the abundance, activity, or diversity of soil organisms is generally viewed as positive (Bünemann et al., 2006), maximizing overall microbial activity or niche saturation which results in competitive exclusion of pathogens, higher levels of nutrient cycling and increased community stability (**Figure 2**). In that regard, the main microbial strategy to enhance the rhizosphere microbiome include the direct inoculation of microorganisms, focussing on co-inoculation with several strains or mixed cultures of arbuscular mycorrhizal fungi (AMF), ectomycorrhizal fungi (ECM), PGPR and endophytes, enabling combined niche exploitation, cross-feeding, enhancement of one organism’s colonization ability, modulating plant growth, achieving niche saturation and competitive exclusion of pathogens (Ping and Boland, 2004; Bünemann et al., 2006; Ryan et al., 2009). Equally important as the recruitment of the adequate microbiome for the plant, is the activation of its specific functions. Quorum sensing (QS) is the mechanism used to regulate distinct microbial activities (biofilm formation, virulence, symbiosis, antibiotic production, conjugation) and is essential for within-species communication as well as for the crosstalk between species which defines if the relationship with the host plant is synergic or antagonist (Soto et al., 2006; Hartmann et al., 2009; Straight and Kolter, 2009).

Enhancing the rhizosphere and root endosphere microbiome often leads to an improvement of beneficial plant functional traits as the microbes are able to expand the plant biochemical capabilities or alter existing pathways (**Table 1**). For instance, PGPR promote plant growth by acting as biofertilizer and entering in symbiosis with their host plants, endosymbiotic rhizobia (*Bradirhizobium, Mesorhizobium, Rhizobium, Sinorhizobium*, etc) and free-living diazotrophs (*Azospirillum, Acetobacter, Herbaspirillum, Azoarcus*, and *Azotobacte*r, etc) fix atmospheric nitrogen, mycorrhiza recover N from NH_4_ and NO_3_ (Toussaint et al., 2004; Adesemoye et al., 2008; Mabood et al., 2008; Ryan et al., 2009)_,_ and phosphate solubilizing bacteria, AMF, ECM, and siderophore producers increase availability of many nutrients such as phosphorous (P), iron (Fe), cooper (Cu), Cadmiun (Cd), and zinc (Zn) (Savka et al., 2002; Mabood et al., 2008). Rhizobacteria also act as biocontrol agents, for instance, Pseudomonads, *Bacillus*, and *Streptomyces* produce antibiotics (DAPG, phenazine, hydrogene cyanide, oligomycin, etc), bacteriocines (Nisin) and antifungal compounds (pyoluteorin, phenazines, and phoroglucinols; Ping and Boland, 2004; Mabood et al., 2008; Paulin et al., 2009; Ryan et al., 2009).

Another strategy is to induce plant metabolic activities by modulating phytohormone synthesis by microbes (phytostimulation). As we mention before, microorganisms are capable of altering plant physiological pathways since they are able to produce all plant hormone identified to date (Friesen et al., 2011) or synthetize compounds that can mimic their actions (**Table 1**). Using microbes to exploit the plant hormonal system could improve plant growth and root development, leading to higher yields. Phytohormones produced by microorganisms such as auxins (indole-3-acetic acid), gibberelins and citokinins mirror the action of jasmonic acid (JA) which is critical for plant defense against herbivory, plant responses to poor environmental conditions (abiotic and biotic stress tolerance), regulation of signals exchange and nodulation (Hause and Schaarschmidt, 2009) and is involved in the signaling pathway against necrotrophic pathogens (Stein et al., 2008). Crosstalk mediated by salicylic acid, JA and ethylene activate plant SAR and induce systemic resistance (ISR) reducing phytotoxic microbial communities (Ping and Boland, 2004; van Loon et al., 2006; Mabood et al., 2008). Production of 1-aminocyclopropane-1-carboxylate (ACC) deaminase by rhizosphere microorganism is another characteristic that can have a high impact on plant health as this enzyme degrades the ethylene precursor ACC, thereby reducing ethylene levels in the plant. When present in high concentrations ethylene can lead to plant growth inhibition or even death, but in lower amounts ethylene can also help the plant respond to a wide range of environmental stresses (Ryan et al., 2009; Glick, 2014).

Inoculation of recombinant strains is another strategy to enhance plant performance. In some cases, recombinant strains can resolve problems related to rapid decrease in population density and short persistence (Ryan et al., 2009), and as reported by Taghavi et al. (2005), could result in the enhancement of many members of the endogenous population by the transmission of genetic information via horizontal gene transfer (HGT). Even though very promising, the release of recombinant strains to the environment needs to be thoroughly assessed in order to evaluate the potential risks associated. To maximize the effects of inoculations, the disruption of existing microbial communities by fungicide application, crop rotation or tilling can be used to favor the selection of the appropriate microbiome for specific crops in order to establish biological functions in the rhizosphere (O’Connell et al., 1996; Savka et al., 2002; Bakker et al., 2012; **Table 1**). It is also essential to understand the evolution, organization, and structure of the rhizosphere microbial community throughout plant developmental stages and the way they naturally manipulate the composition of the rhizosphere microbiome, promoting, for instance, suppressive soils (Baker and Cook, 1974; Ryan et al., 2009; Mendes et al., 2011) or particular microbial functions in the rhizosphere like nutrient cycling or resistance to abiotic stress. In addition to specific microbial taxa or functions, community-wide characteristics can also be the target of microbiome engineering efforts as rhizosphere microbiome richness and evenness is linked to higher resilience to disruption, stress, and diseases. Increased microbial richness often results in greater community-level trait diversity and/or functional redundancy, which leads to more consistent functioning across variable environments (Loreau et al., 2001) Because rare members of the microbiome may be unable to effectively perform important functions, high evenness of the microbiome is also very important (**Figure 2**; Bünemann et al., 2006; Badri and Vivanco, 2009; Bakker et al., 2012; Qiu et al., 2014).

### The Plant Route

Plant-based strategies to improve plant productivity through the selection of a more adapted microbiome include the manipulation of plants characteristics of interest mainly by two different approaches: plant breeding (cultivar selection) and specific genetic modifications (**Table 2**). Using plant breeding to select for a specific microbiome is an interesting avenue, as the technique has mainly focused on improving yields, plant resistance to pests/diseases and other plant physiological traits (Ryan et al., 2009). When microbiome selection was included in plant breeding programs, very specific functions or taxa were targeted. For example, Neal et al. (1970, 1973) used chromosomal substitution between two lines of wheat to improve resistance to root-rot while preserving beneficial populations of rhizosphere bacteria, and Mazzola (2002) compared wheat cultivars for their capacity to stimulate disease suppression by enhancing populations of specific antagonist (Pseudomonads) against *Rhizoctonia solani*.

Choosing a naturally occurring plant species or cultivar with a high capacity to recruit a beneficial microbiome or to promote the “suppressiveness” of soils is an alternative option that has been explored (Neal et al., 1973; Mazzola, 2002; Bakker et al., 2012). For instance, in the rhizosphere of plants growing in contaminated soils, the host plant exudes specialized antimicrobials and signaling molecules (i.e., flavonoids, salicylic acid, and phytoalexins), carbon and nitrogen compounds that promotes the expression of hydrocarbon degradation genes such as the genes coding for alkane hydroxylases (responsible for the aerobic degradation of aliphatic hydrocarbons) and several genes coding for enzymes implicated in the metabolism of aromatic compounds (Yergeau et al., 2014; Pagé et al., 2015). This recruitment was shown to be cultivar-specific, with native cultivar having an increased capacity to recruit beneficial ECM when growing in highly contaminated soils (Bell et al., 2014a), suggesting that native cultivars can better communicate with indigenous soil microorganisms. Accumulating evidence supports the feasibility of creating biotic soil environments that promote root health using selected plant genotypes. Ellouze et al. (2013) showed in the semiarid grasslands of North America, certain chickpea cultivars can select a more beneficial microbiome for the subsequent wheat plants and were associated with the antagonist species *Penicillium canescens*.

Using plants as selective agents to enrich beneficial microbial functions in soil implies the inclusion of other variables such as soil type and properties: different soil types not only shape the microbial communities, but also impact plant physiology, which in turn could alter interactions with soil microbes. The creation of genetically modified plants with enhanced ability to harbor particular exudation patterns to change soil properties have already been investigated (**Table 2**). Li et al. (2005); Gévaudant et al. (2007), and Yang et al. (2007) have worked to manipulate rhizosphere pH using transgenic lines of *Nicotiana tabacum* and *Arabidopsis* plants, transformed to over express a modified H^+^ATP-ase protein (*PMA4* in tobacco and *AVP1 pyrophosphatase* in *Arabidopsis*) generating phenotypes such as increased H+-eﬄux from roots, more acidic rhizosphere, improved growth at low pH, improved salinity resistance (tobacco lines), plant mineral nutrition (P mineralization), and exhibiting enhanced resistance to water deficits (*AVP1*).

Plants can also be genetically modified to alter soil organic anion eﬄux and transportation from roots by (1) engineering plants with a greater capacity to synthesize organic anions or (2) engineering plants with a greater capacity to transport organic anions out of the cell. The organic anions malate and citrate have been studied as they are commonly release in response to nutrient deficiency and mineral stress. Koyama et al. (1999) and Koyama et al. (2000) reported that transgenic plants with higher ability to excrete citrate from the roots grew better on P-limited soil than the wild type, suggesting crop plants with an enhanced ability to use Al-phosphate and therefore an enhanced ability to grow in acid soils and superior Al tolerance. In order to address the toxicity of the Al^3+^ in acidic soils, a very common problem in agriculture, Tesfaye et al. (2001), Anoop et al. (2003), and Delhaize et al. (2007) have also reported the use of transgenic plants *(Medicago sativa, Brassica napus*, and *Hordeum vulgare*) expressing genes coding for ALMT (Al^3+^-activated malate transporter) and MATE (multi-drug and toxin extrusion) membrane proteins, a strategy to improve the P efficiency of plants and Al^3+^ resistance (**Table 2**).

Quorum sensing has been targeted by creating transgenic plants that would be able to mimic or interrupt bacterial QS signals by producing enzymes responsible for their degradation (acylases and lactonases). These modifications allowed these plants to defend themselves more efficiently against some pathogenic bacteria (Savka et al., 2002; Braeken et al., 2008; Ryan et al., 2009; Bakker et al., 2012). Many other studies have focused on the genetic manipulation of plants in order to modify the key exudates to favor the establishment of the desired plant–microbiome (**Table 2**). Genetic engineering provides unique opportunities to modulate plant–microbe signaling, to diversify exudation, to encourage diverse microbiomes or to stimulate beneficial microbial functions in the rhizosphere. However, despite these efforts, large-scale breeding/genetic improvement programs rarely take into account the plant–microbiome signaling channels during the development of new plant lines.

### The Meta-Organism Route

The microbiome and the plant are highly dependent on each other as the microbiome contribute a significant portion of the secondary genome of the host plant, highlighting that the plant an its microbiome might function as a meta-organism or holobiont (Lakshmanan et al., 2014). Taking into account the meta-organism and trying to optimize the whole system instead of each of the part separately is a promising avenue for rhizosphere microbiome engineering. This is the case of the “opine concept” that combined engineering plants to produce specific exudates together with the inoculation of engineered microbes that are able to degrade this substrate, resulting in the colonization of the rhizosphere by a specific population (**Table 3**). It was also observed that opine production by transgenic plants led, in the long term, to the selection of bacterial populations adapted to the rhizosphere that can maintain themselves at high concentrations, even after removal of the transgenic plants (O’Connell et al., 1996; Savka et al., 2002; Ryan et al., 2009). These strategies (using specific metabolic resources) are highly specific, focusing on interactions between, for example, opine-producing plants, and members of the microbiome responsible for functions such as nodulation and *N*-fixation. However, this “opine” approach does not take into account others species that could have important functions or fill niche to reduce pathogen vulnerability.

In order to amplify the spectrum of diversity and ecological services to the crops, another strategy to shift rhizosphere microbiome is crop rotation. This strategy could be optimized by taking into account the whole plant–microbiome metaorganism. Indeed, plants are cultured in turns bringing their associated microbiome and generating beneficial allopathy. Crop history and cultivar selection stimulate specific rhizobacterial populations that complements each other developing a beneficial synergy between the cultures. Sturz et al. (1998) reported evidence for the role of bacterial endophytes resulting from the intercrop alternation of red clover and potato promoting plant growth and yield in the potato crops. Mazzola (2007) stated that most common and effective scheme to modify the rhizosphere has been the use of crop rotation. As increased plant diversity can enhance microbial community biomass, mixed cropping systems will generate a more diverse microbial community and thus should be more resilient to pathogen invasion. Disease control is achieved as the plant host for certain pathogen is absent resulting in the diminished viability of this pathogen. Some other advantages of these rhizosphere microbiome engineering approaches are an increase of soil organic carbon, a higher level of nutrients cycling and an improvement of physico-chemical soil characteristics (O’Connell et al., 1996; Sturz et al., 1998; Bakker et al., 2012).

Phytoremediation is another application where harnessing the plant–microbiome holobiont could significantly improve processes (Bell et al., 2014b). In the rhizosphere of contaminated soils, microbes increase the recycling and dissolving of mineral nutrients and the synthesis of amino acids, vitamins, auxins, and gibberellins that stimulate plant growth. These highly competitive populations seem to be selected by the host plant via exudation of specialized antimicrobials and signaling molecules (e.g., flavonoids, salicylic acid, and phytoalexins), carbon and nitrogen compounds, resulting in the degradation or transformation of contaminants due to both increased microbial activity and plant intervention (Marihal and Jagadeesh, 2013; Yergeau et al., 2014). Microorganisms also facilitate the uptake of contaminants and plant resistance to pollutant stress (Taghavi et al., 2005; Nadeem et al., 2013; Bell et al., 2015).

Finally, we should briefly mention organic agricultural inputs as a complementary strategy not included in the abovementioned categories (microbe, plant, or meta-organisms), but that also results in the modification of the rhizosphere microbiome. Organic agriculture aims at limiting or preventing the exposure of plants, microbes, and humans to unnecessary hazards such as pesticides, herbicides, insecticides, and fungicides. Organic fertilizer such as animal manure, biosolids, and compost has been proposed as a resource to amend crops but some disadvantage should be taken into account, namely, increase salinity, presence of active therapeutic agents (manure and sludge waste), heavy metals such as zinc, cooper, and cadmium (industrial biosolids) and residues of synthetic molecules like pesticides, herbicides etc, (green wastes or compost; **Table 3**). An important aspect to consider when applying any agricultural inputs is to target the increase in the levels of soil organic matter that in turn, will also increase soil biological activity (Savka et al., 2002; Bünemann et al., 2006; Mazzola, 2007).

## Conclusion and Outlook

The microbiome is emerging as a fundamental plant trait, resulting in beneficial or detrimental effects on plant growth, health, productivity, and functions. This delicate balance is controlled by complex chemical signals interplay between the plant and its microbiome. Further research aiming at understanding this interplay at the community level is needed to fully understand the factors controlling microbiome assemblage and its feedback to the plant host. New ‘omics tools will undoubtedly help attaining that goal, but at the same time further efforts to cultivate the rhizosphere microbiome will also be needed to reach a deeper mechanistic understanding of it. Based on the engineering efforts detailed in this contribution, further research will hopefully result in methods to purposefully, reliably, and sustainably engineer plant–microbiomes. A full optimization of the plant–microbiome meta-organism should result, among others, in a more sustainable agriculture, reduced greenhouse gas emissions, and increased rates of soil decontamination.

## Conflict of Interest Statement

The authors declare that the research was conducted in the absence of any commercial or financial relationships that could be construed as a potential conflict of interest.
